# Curcumin Promotes the Recovery of Motor Function After Brachial Plexus Avulsion Injury in Rats

**DOI:** 10.1002/brb3.70728

**Published:** 2025-08-04

**Authors:** Sijing Li, Bing He, Guijuan Zhou, Lin Wu, Xuanwei Wen, Limin Deng, Shudong Lin, Guozhi Liu, Shuangxi Chen, Zijian Xiao

**Affiliations:** ^1^ Department of Neurology, Multi‐Omics Research Center for Brain Disorders, The First Affiliated Hospital, Hengyang Medical School University of South China Hengyang Hunan China; ^2^ Clinical Research Center for Immune‐Related Encephalopathy of Hunan Province (The First Affiliated Hospital), Hengyang Medical School University of South China Hengyang Hunan China; ^3^ Department of Clinical Laboratory Medicine, The First Affiliated Hospital, Hengyang Medical School University of South China Hunan China; ^4^ Department of Neurology Chenzhou, No.1 People's Hospital Chenzhou China; ^5^ Department of Neurology, The Second Affiliated Hospital, Hengyang Medical School University of South China Hengyang Hunan People's Republic of China

**Keywords:** Brachial plexus injury, curcumin, inflammation

## Abstract

**Background and Purpose:**

Brachial plexus root avulsion (BPRA) often results in the loss of upper limb motor function. Curcumin (CUR) has been proven to have neuroprotective properties in various neurological disorders due to the effects of anti‐oxidative stress and anti‐inflammation. Therefore, in this study, we focused on the effect of CUR on the recovery of motor functions in rats after BPRA.

**Methods:**

Adult male Sprague‐Dawley rats were used to build the BPRA and reimplantation model and randomly divided into two groups: the NS group (treated with saline) and the CUR group (treated with 40 mg/ml CUR), with 10 rats in each group. After conducting the Terzis grooming test (TGT) to assess the recovery of motor function, the anterior horn of the spinal cord was collected for detecting the inflammatory responses using Western blot, the musculocutaneous nerves were collected for detecting the motor neuron survival and myelination using Luxol fast blue (LFB) staining or real‐time quantitative PCR (qRT‐PCR), and the biceps brachii were collected for detecting muscle atrophy using hematoxylin and eosin (H&E) staining.

**Results:**

CUR can significantly enhance the motor recovery in rats following BPRA, reduce inflammation in the anterior horn of the spinal cord, improve the motor neuronal survival and axonal remyelination in musculocutaneous nerves, alleviate muscle atrophy.

**Conclusion:**

CUR promotes the recovery of motor function in rats after BPRA by inhibiting inflammation, reducing motor neuron death, promoting axonal remyelination, and reducing muscle atrophy, thus laying a foundation for the treatment of BPRA with CUR.

## Graphical abstract

CUR promotes the recovery of motor function in rats after BPRA by inhibiting inflammation, promoting axonal remyelination, and reducing muscle atrophy, laying a foundation for the treatment of BPRA with CUR.

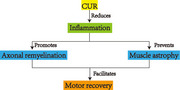

## 1 Introduction

The brachial plexus, composed of the nerve roots from the fifth to the eighth cervical vertebrae (C5‐C8) and the first thoracic vertebra (T1) (Boulanger et al. 2013), is particularly vulnerable to damage during high‐energy impact events such as car accidents (Huang et al. 2022). Brachial plexus injury can lead to the avulsion of spinal roots with an incidence of 21%, resulting in the death of motor neurons (MNs) and degeneration of axons, which in turn causes muscle atrophy and complete paralysis of the upper limbs (Smith et al. 2019; Zhao et al. 2020), severely impacting the quality of life of patients (Giuffre et al. 2010). Brachial plexus root avulsion (BPRA) has been explored by numerous researchers through various alternative treatment methods in animal models, including local application of neurotrophic factors (Xian et al. 2023) and cell transplantation strategies (Jin et al. 2015). However, the effectiveness and clinical applicability of these methods have shown several limitations (Jin et al. 2015). BPRA not only causes initial damage such as axonal injury and muscle atrophy but also triggers a series of complex secondary injury cascades, including inflammation and cell death. These secondary responses, in turn, exacerbate the initial damage, making recovery more difficult (Ham and Leipzig 2018). Therefore, the drugs with anti‐inflammatory and anti‐apoptotic properties may be potential candidates for the treatment of BPRA.

Attempts to search for the candidates that may promote the recovery of motor function following BPRA have been made to counteract the MN death induced by anti‐inflammation. One of these candidates is curcumin (CUR), a polyphenolic compound extracted from turmeric with the most important active pharmacological properties. It promotes the development of synapses in rat hippocampal neurons by inhibiting GSK‐3β and activating β‐catenin (Zhang et al. 2024). Furthermore, CUR can also slow the progression of disease and alleviate the symptoms of experimental autoimmune encephalomyelitis (Amiri et al. 2023), while also inhibiting the inflammatory response in ischemic stroke (Du et al. 2023). CUR can also prevent neurodegeneration by inhibiting neuroinflammation in Parkinson's disease (Cai et al. 2025). CUR can ameliorate astrocyte inflammation in cuprizone‐induced mice (Zhang et al. 2025). Delivery of CUR nanoparticles can improve neurological deficits in mice with intracerebral hemorrhage by inhibiting neuroinflammation (Duan et al. 2024).

Given the pharmacological activities of CUR in neuroprotection, we are particularly interested in whether it can facilitate functional motor recovery after BPRA. In this study, we reported that CUR can mitigate inflammation, promote myelination of musculocutaneous nerve axons, and prevent muscle atrophy, significantly enhance motor function recovery in rats following BPRA.

## 2 Materials and Methods

### 2.1 Animals

Adult male Sprague Dawley rats weighing 210–230 g and purchased from Changsha Tianqin Biotechnology Co., Ltd. were maintained on a 12‐h light/12‐h dark cycle at 18–22°C and humidity at 50%–60% in the Experimental Animal Department of the University of South China. Each rat was housed with equal access to food and water. All procedures conducted on the animals were approved by the Animal Ethics Committee of the University of South China (Approval No. 2022LL0102003).

### 2.2 BPRA Model and Groupings

**FIGURE 1:**
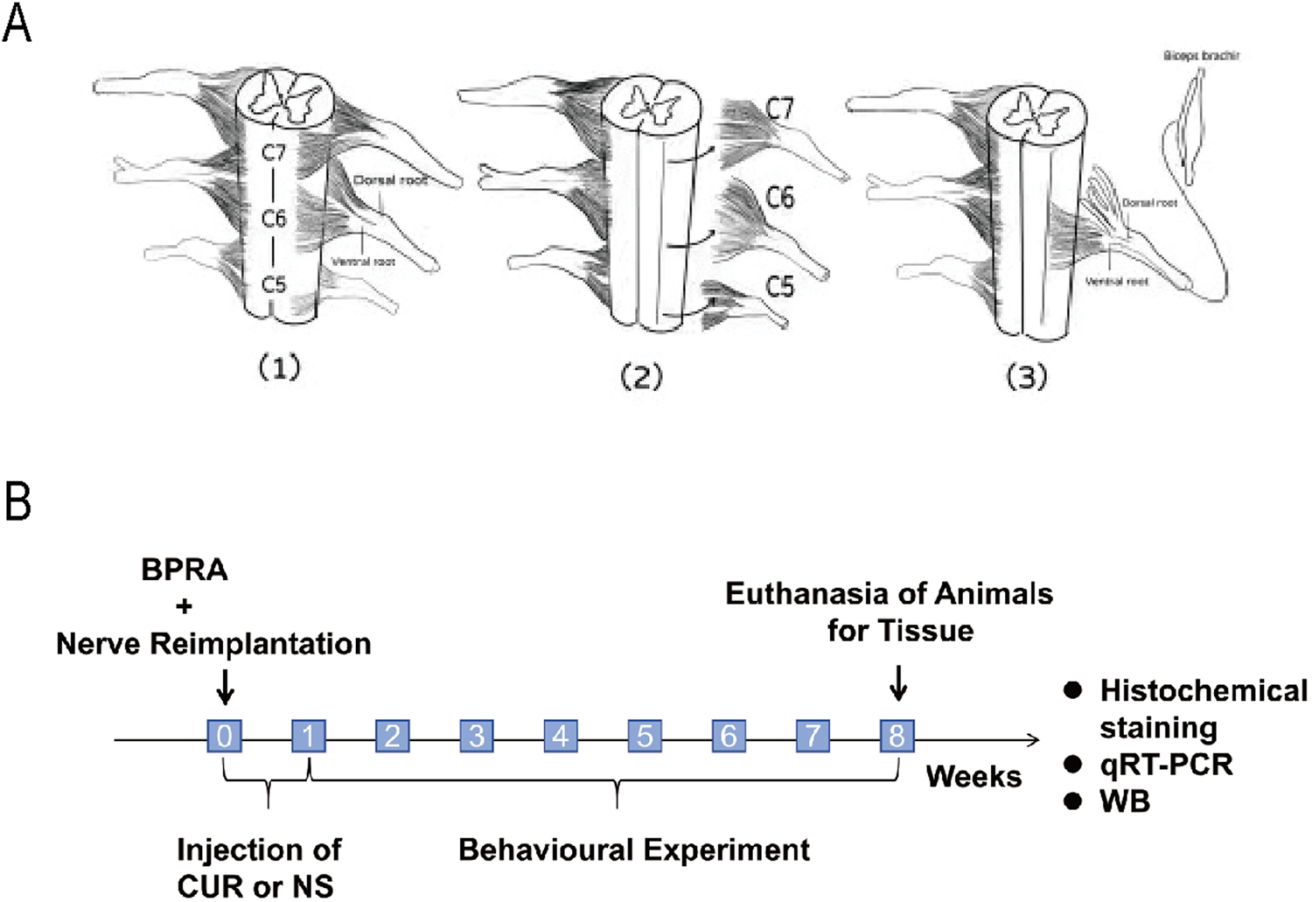
**BPRA and reimplantation rat model. (A)** (1) Schematic diagram of the C5–C7 spinal cord segments before surgery. (2) The ventral and dorsal roots of the right C5–C7 spinal cord segments are torn off. (3) The ventral root of the right C6 spinal cord segment is reimplanted into the spinal cord and **(B)** Schematic diagram of experimental procedure.

As shown in Figure 1A and1B, the surgical procedure was performed as previously described (Chen et al. 2021; Wu et al. 2023). Briefly, the rats are anesthetized with an intraperitoneal injection of 10% chloral hydrate (5 µL/kg), and then, the rats are positioned prone on a custom‐made dissection table. Next, a midline incision is made in the nape with ophthalmic scissors, and the subcutaneous fascia and muscles are cut layer by layer. Under a dissecting microscope, the segments are accurately located based on the position of the thoracic 2 (T2) spinous process. The laminae from C4 to C7 are then removed with bone rongeurs, and the dura mater is incised to expose the C5–C7 nerve roots, which are bluntly avulsed using forceps, and the ventral root of C6 was reimplanted into their original spinal segments. After the surgery, the rats were returned to their cages for continued care.

The BPRA rats were randomly divided into two groups: the NS group and the CUR group, with ten rats in each group. In the CUR group, rats received a subcutaneous injection of 500 µL CUR at a concentration of 40 mg/ml daily for 7 days near the injury site. In the NS group, rats were injected subcutaneously with an equivalent volume of saline.

### 2.3 Terzis Grooming Test (TGT)

TGT was conducted weekly to assess the recovery of elbow flexion function in the right forelimb of the rats and was performed as previously described (Chen et al. 2021; Wu et al. 2023). A small spray bottle is used to evenly distribute pure water onto the rats' head and face, prompting them to groom their head and face with both forelimbs. Normally, rats will flex their elbows and raise their forelimbs to wipe away the water droplets from their head and face in a top‐down motion. The scores are recorded; the highest score of the movement within 3 min is described as follows: 0 points, no response of the affected limb when lifted; 1 point, the affected limb's elbow flexes and the forepaw is lifted but does not reach the nose; 2 points, the affected limb's elbow flexes and the forepaw reaches the nose; 3 points, the affected limb's elbow flexes and the forepaw exceeds the nose but does not exceed the eyes; 4 points, the affected limb's elbow flexes and the forepaw exceeds the eyes but does not reach the ears; 5 points, the affected limb's elbow flexes and the forepaw reaches the ears.

### 2.4 Tissue Preparation

Post‐surgery for 8 weeks, rats were anesthetized using chloral hydrate, and tissues were subsequently harvested for further analysis.

For Western blot analysis, the anterior horns of the right side of the C5–C7 spinal segments were dissolved in RIPA buffer containing 100 µL of 1% PMSF. After homogenization, the tissues were centrifuged at 13,000 g for 20 min at 4°C, and the supernatant was collected.

For qRT‐PCR analysis, total RNA was extracted from musculocutaneous nerve tissues using TRIzol reagent following the manufacturer's previously described protocol.

For histological staining, rats were perfused with saline through the heart, followed by fixation with 4% paraformaldehyde (PFA). The musculocutaneous nerves and biceps brachii were dissected. After fixing the tissues in 4% PFA at 4°C for 24 h, they were transferred to PBS supplemented with 15% and 30% sucrose and stored at 4°C for 24 h. After being sunk, the musculocutaneous nerves and biceps brachii tissues were cut into sections at a thickness of 10 µm on a sliding microtome (LEICA CM1950, Leica).

### 2.5 Western Blot Analysis

Western blot was conducted as previous studies (Tan et al. 2022;Wan et al. 2022;Chang et al. 2023). Protein samples were loaded, and the electrophoresis was conducted at 90 V for 20 min through the stacking gel and 160 V for 120 min through the resolving gel. During the transfer step, the gel and NC membrane were assembled in the specified order, and the transfer was carried out at 300 mA constant current for 2 h. The membrane was stained with Ponceau Red for lane marking. The membrane was incubated with primary and secondary antibodies listed in Table 1 in 3% BSA‐TBST, followed by washing with 0.1% TBST. Finally, the NC membrane was developed using Chemiluminescence (ECL) substrate, and the images were obtained.

**TABLE 1:** Primary antibodies for Western blot.

Name	Code	Source	Dilution ratio	Company
SIRT1	ab110304	Mouse	1:1000	Abcam
nNOS	ab76067	Rabbit	1:1500	Abcam
P‐P65	ab86299	Rabbit	1:3000	Abcam
β‐actin	66009‐1‐Ig	Mouse	1:5000	Proteintech

### 2.6 Quantitative Real‐time PCR (qRT‒PCR)

QRT‒PCR was performed as previously described (Duan et al. 2024). The concentration of the RNA obtained was measured using the NanoDrop 2000 instrument (Thermo Scientific, Rockford, IL, USA). 1 mg of total RNA was reverse transcribed into first‐strand cDNA using the PrimeScript RT reagent kit with gDNA Eraser (TaKaRa). Then, this cDNA was used as a template for PCR amplification with SYBR Green Master Mix (Solarbio, Beijing, China) and analyzed on an Exicycler 96 Real‐Time PCR System (ABI 7500, Applied Biosystems). The sequences of the qRT‐PCR primers are listed in Table 2. Finally, the specificity of the PCR products was verified by melt curve analysis, and gene expression levels were calculated using the 2‐^ΔΔCT^ method.

**TABLE 2:** Primer sequences for qRT‐PCR.

Name	Toward (5’–3’)		Sequences (bp)
GAP43	Forward	TGCCCTTTCTCAGATCCACT	188
	Reverse	AAAACTCGCCATAACAACACC	
L1CAM	Forward	GGGACCTACAGCCTGACACCAAA	158
	Reverse	AGCACTGACAAAGGCGATGAACCA	
NGRF	Forward	ACGACCAGCAGACCCATACGC	131
	Reverse	ATGTCGCCAGGTATCCCCGTT	
HMGCR	Forward	ACATACTGGACTGAAACACGGGCAT	206
	Reverse	AGAACACGGCACGGAAAGAACCAT	
POU3F1	Forward	ACATGGCCCAGCACTACCAGA	199
	Reverse	ATCTCTCCCCTTCTCCAGTTCG	
β‐actin	Forward	ACATCCGTAAAGACCTCTATGCC	223
	Reverse	TACTCCTGCTTGCTGATCCAC	

### 2.7 Histochemical Staining

#### 2.7.1 Luxol Fast Blue (LFB) Staining

LFB staining was performed as previously described (Chen et al. 2019; Chen et al. 2020). Myelin Stain A was preheated to 65°C for 30 min prior to use. Sections of the musculocutaneous nerve were then stained with Myelin Stain A for 4 h at the same temperature. Subsequently, these sections were treated with Myelin Stain B for 5 s, followed by a 10‐s immersion in Myelin Stain C.

#### 2.7.2 Hematoxylin and Eosin (H&E) Staining

H&E staining was performed as previously described (Chen et al. 2021). The biceps brachii sections were initially stained with hematoxylin for 3–5 min to highlight nuclei, following a brief immersion in the hematoxylin solution for 2–5 s. The sections were then stained with eosin for 5 min to ensure cytoplasmic delineation. Subsequently, the tissue underwent dehydration 3 times using absolute ethanol, each for a duration of 5 min.

Images from LFB staining and H&E staining were captured using an MBF Nikon light microscope. The number of nerve fiber counts, nerve diameters, muscle fiber diameters, and fibroblast nuclei counts were quantified using ImageJ 5.0 software.

### 2.8 Statistical Analysis

Data are presented as mean ± standard deviation (SD). Statistical analyses were performed using GraphPad Prism 6.0 software following either two‐way ANOVA or Student's *t*‐test followed by a post‐hoc Bonferroni test. A *p‐*value of less than 0.05 was considered statistically significant.

## 3 Results

### 3.1 CUR Treatment can Promote the Recovery of Impaired Forelimb Motor Function in Rats After BPRA

**FIGURE 2:**
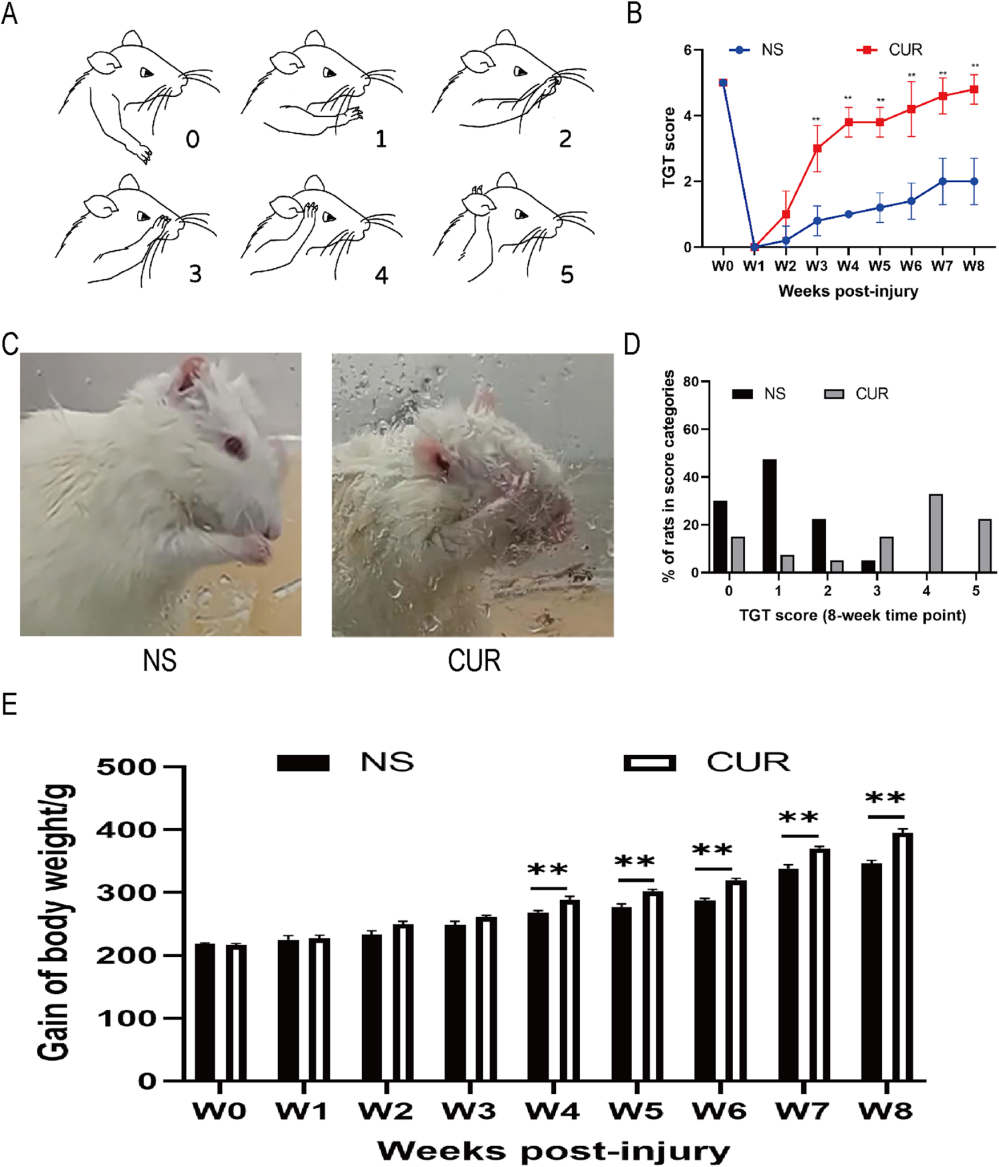
**CUR treatment can promote the recovery of impaired forelimb motor function in rats after BPRA. (A)** Position of the forelimbs and corresponding grooming test scores: 0, no movement; grade 1, elbow flexion but unable to touch the nose; grade 2, elbow flexion and able to touch the nose; grade 3, elbow flexion and able to reach below the eye; grade 4, elbow flexion and able to reach the eye; and grade 5, elbow flexion and able to reach the ear or back of the ear, **(B‐D)** The averaged TGT score was increased by the treatment of CUR, and **(E)** The average gain of body weights was increased after the treatment of CUR. **p < 0.01, *p < 0.05 (*n* = 5).

To investigate whether CUR could help rats recover forelimb motor function, we conducted a weekly TGT to assess the motor function recovery of their injured limbs. As shown in Figures 2A‐D, the corresponding scores were obtained based on the position reached by the forelimb. The experimental data show that in the first week after surgery, rats treated with CUR and the control group (rats injected with normal saline, NS group) were unable to move their forelimbs, scoring 0, indicating successful surgery and complete loss of motor function in the rat forelimbs. Subsequently, we continued to monitor the recovery of the rats. two‐independent‐samples *t*‐test showed that the TGT score of the CUR group was significantly higher than that of the NS group at 3, 4, 5, 6, 7 and 8 weeks after operation. (W3: *t* = 5.880, *p* = 0.0004; W4: t = 14.00, *p* < 0.0001; W5: *t* = 9.192, *p* < 0.0001; W6: *t* = 6.261, *p* = 0.0002; W7: *t* = 6.500, *p* = 0.0002; W8: t = 7.483, *p* < 0.0001) (Figure 2B).

In addition, all rats were given consistent amounts of food and water, which were placed in the same locations. Rats with impaired motor abilities also showed diminished capacity to consume food. Therefore, we also monitored changes in the rats' weight as another auxiliary indicator of motor function recovery. A two‐independent‐samples *t*‐test showed that the body weight of rats in the CUR treatment group was significantly higher than that in the NS group at 4–8 weeks after the operation. (W4: *t* = 5.298, *p* = 0.0061; W4: *t* = 5.298, *p* = 0.0061; W5: *t* = 7.181, *p* = 0.0020; W6: *t* = 13.57, *p* = 0.0002; W7: *t* = 7.821, *p* = 0.0014; W8: *t* = 7.821, *p* = 0.0014) (Figure 2E).

### 3.2 CUR Treatment can Alleviate Inflammatory Responses in Rats After BPRA

**FIGURE 3:**
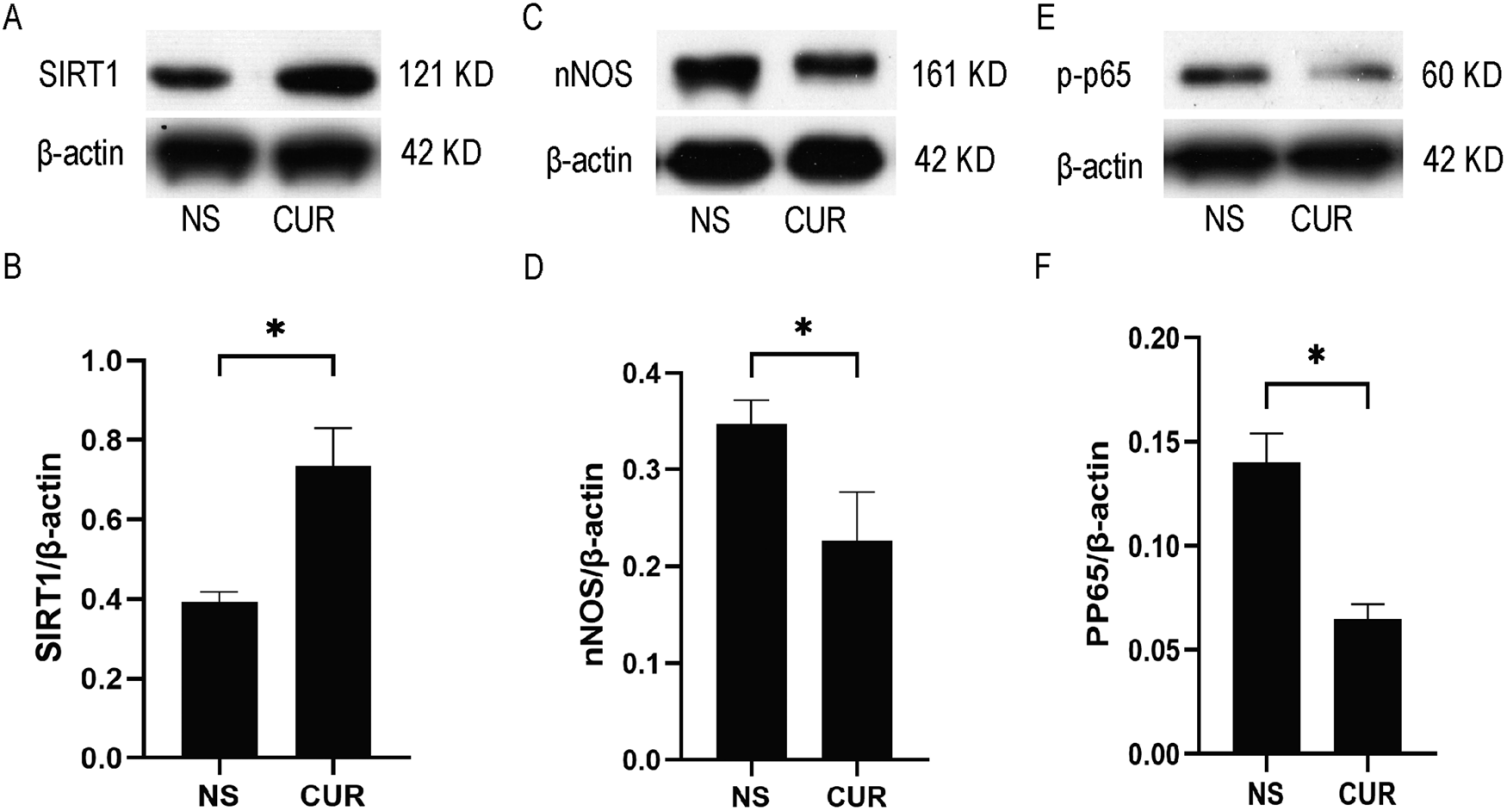
**CUR treatment can alleviate inflammatory responses in rats after BPRA**. The protein levels of **(A‐B)** SIRT1 were increased, and **(C‐D)** nNOS, and **(E‐F)** P‐P65 were decreased in the anterior horn of the spinal cord of BPRA rats after the treatment of CUR. *p < 0.05 (*n* = 3).

To investigate whether CUR can suppress inflammation in rats after BPRA, Western blot was performed to measure the levels of sirtuin 1 (SIRT1), nNOS (neuronal nitric oxide synthase), and P‐P65 in the anterior horn. Independent sample *t*‐test results showed that, compared to the NS group, the level of SIRT1 in the affected side of the anterior spinal cord in the CUR group was significantly increased (*t* = 5.999, *p* = 0.0039), while the levels of nNOS (*t* = 3.694, *p* = 0.0210) and P‐P65 (*t* = 6.708, *p* = 0.0215) were significantly decreased (Figures 3A–C).

### 3.3 CUR Treatment can Reduce Neuronal Death and Promote the Remyelination in the Musculocutaneous Nerve of Rats Following BPRA

To explore the effects of CUR on the morphology of the musculocutaneous nerve in rats after BPRA, we observed the diameter of the musculocutaneous nerves and the number of LFB‐positive axons and used qRT‐PCR to measure the mRNA levels of GAP‐43, L1CAM, HMGCR, NNGFR, and POU3F1 in the musculocutaneous nerves of the affected limbs in rats at 8 weeks post‐surgery.

**FIGURE 4:**
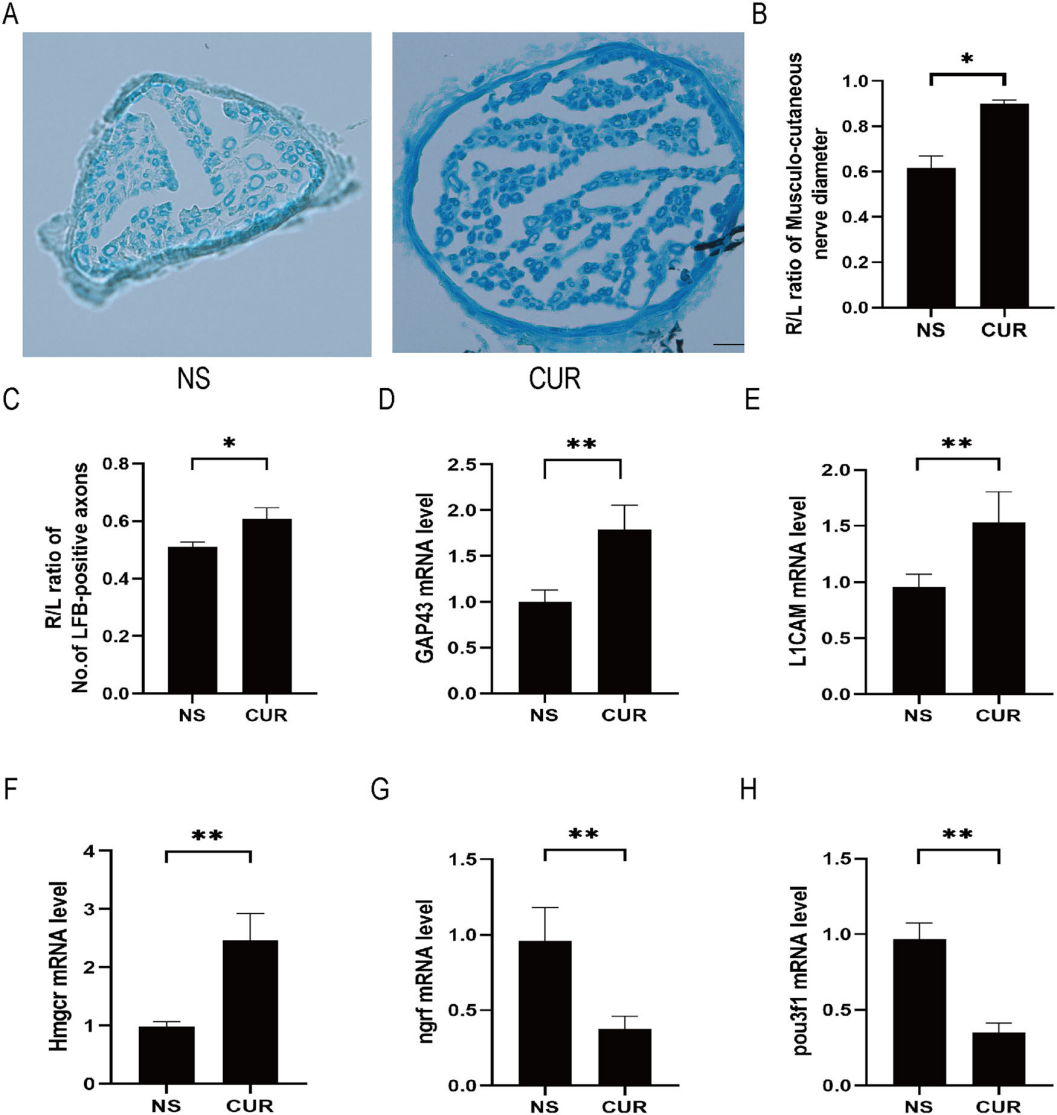
**CUR treatment can reduce neuronal death in the musculocutaneous nerve of rats following BPRA. (A)** Representative images of LFB staining of the musculocutaneous nerve were shown. Scale bar, 100 µm, **(B)** The diameter of the musculocutaneous nerve, **(C)** The number of LFB‐positive axons was increased after the treatment of CUR. Growth‐related genes **(D)** GAP‐43 and **(E)** L1CAM were increased after the treatment of CUR. Remyelination‐related gene **(F)** HMGCR was increased, and demyelination‐related genes, **(G)** NGFR, and **(H)** POU3F1 were decreased after the treatment of CUR in the musculocutaneous nerve. **p < 0.01, *p < 0.05 (*n* = 3).

Independent sample t‐test results showed that, compared to the NS group, the diameter of the musculocutaneous nerve in the CUR group was significantly increased (*t* = 3.912, *p* = 0.0174); compared to the NS group, the number of LFB‐positive axons in the CUR group was significantly higher (*t* = 8.923, *p* = 0.0009) (Figures 4A–C).

As shown in Figures 4D–H, compared to the NS group, the relative expression levels of the axonal growth‐associated protein genes GAP‐43 (*t* = 7.963, *p* < 0.0001) and L1CAM (*t* = 5.882, *p* < 0.0001) and the myelination‐associated gene HMGCR (*t* = 9.648, *p* < 0.0001) are increased in the CUR group, while the expression levels of the differentiation‐associated genes NGFR (*t* = 7.266, *p* < 0.0001) and POU3F1 (*t* = 14.87, *p* < 0.0001) decreased in the musculocutaneous nerves of the affected limb.

### 3.4 CUR Treatment Can Improve Muscle Atrophy in Rats After BPRA

To determine the extent of muscle atrophy after surgery, the biceps brachii of the injured and intact forelimbs were weighed, and the right/left biceps brachii weight ratio was calculated. Then, histopathological changes in the biceps tissues were evaluated using H&E staining.

**FIGURE 5:**
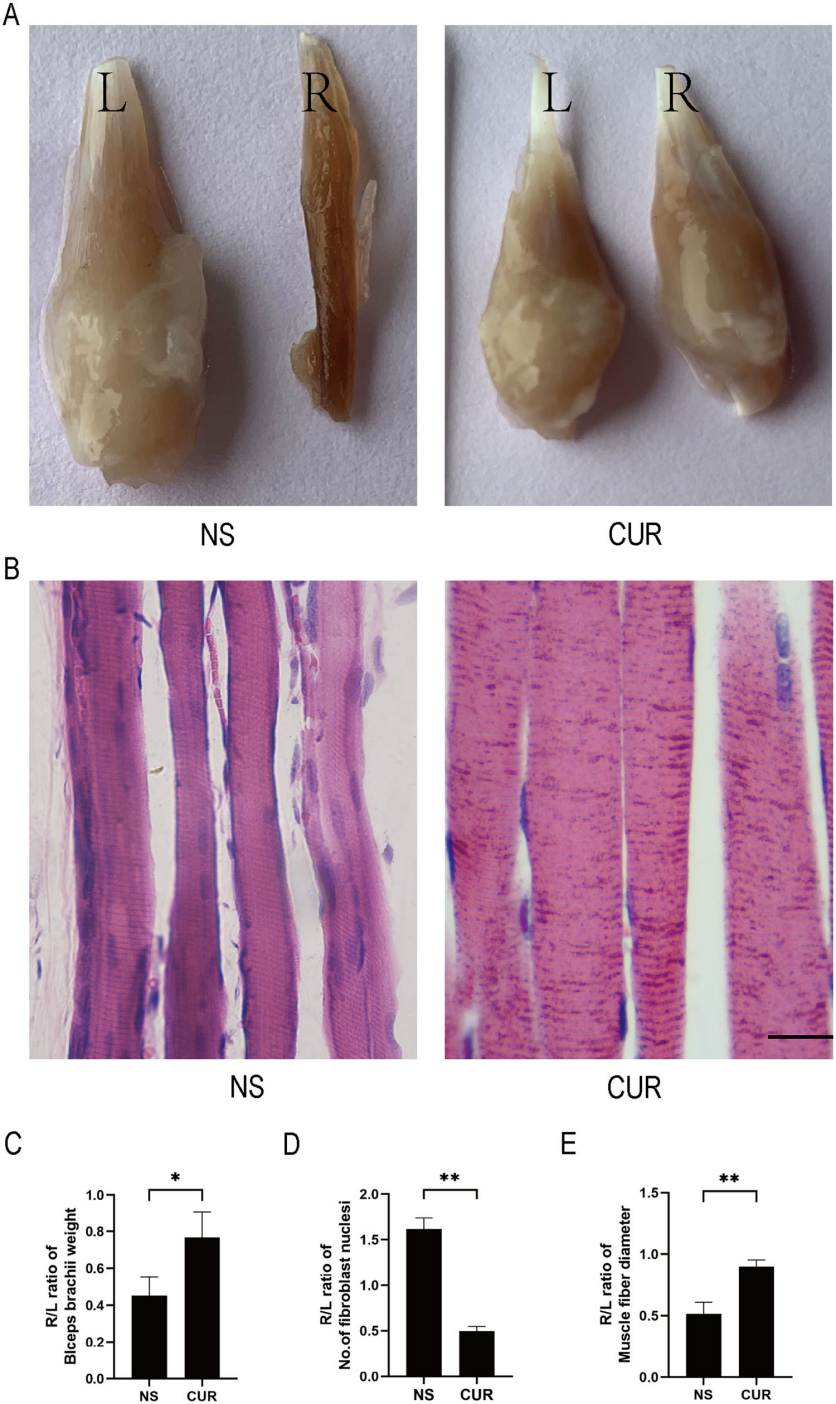
**CUR treatment can improve muscle atrophy in rats after BPRA. (A)** Representative images of the biceps brachii of rats were shown, **(B)** Representative images of H&E staining of the right biceps brachii tissues were shown. Scale bar, 100 µm, **(C)** The weight of biceps brachii was increased after the treatment of CUR, **(D)** The number of fibroblast nuclei of biceps brachii was decreased after the treatment of CUR, and **(E)** The diameter of the fiber of the biceps brachii was increased after the treatment of CUR. *p < 0.05 (*n* = 3).

As shown in Figures 5A and C, the weight of the injured side biceps muscle was significantly increased in the CUR group compared with the NS group (*t* = 3.627, *p* = 0.0110). As shown in Figures 5D, E and F, compared with the NS group, rats in the CUR group had larger muscle fibers (*t* = 6.101, *p* = 0.0037), clearer nuclei of the myocytes, and significantly fewer fibroblasts (*t* = 14.30, *p* = 0.0001).

## 4 Discussion

In previous studies, we have explored the effects of artemisinin (Chen et al. 2021), berberine (Chen et al. 2020), neuregulin‐1 (Chen et al. 2019), Edaravone (Li et al. 2025), and acetylglutamine (Wu et al. 2023) on motor function recovery following BPRA. In this study, we further demonstrate that CUR can significantly enhance functional recovery after BPRA via mitigating inflammation, promoting myelination in musculocutaneous nerve axons, and preventing muscle atrophy, laying the solid foundation for CUR to be a novel candidate for the treatment of brachial plexus injury.

CUR has shown positive effects in the treatment of motor system diseases such as Parkinson's disease (De Guzman et al. 2022) and amyotrophic lateral sclerosis (D'Antona et al. 2021). At present, neurological functions are mostly determined to evaluate the degree of lesion to detect the effect of potential strategies. In this study, we observed that CUR can effectively restore motor function in the affected upper limb in rats following BPRA.

An increasing amount of evidence indicates that inflammation plays crucial roles in brachial plexus injuries (Chen et al. 2019; Huang et al. 2021; Wang et al. 2023). The inflammation intensifies the cascade of responses, inducing widespread degenerative necrosis of spinal motor neurons and inhibiting the regeneration of axons and dendrites, ultimately severely impairing the regeneration and functional recovery of nerve axons (Huang et al. 2021).SIRT1, a neuroprotective protein regulating multiple physiological functions such as metabolism and immune responses (Yang et al. 2022; Patra et al. 2023; Shen et al. 2024). In spinal neurons, nNOS is the predominant subtype of NOS and is closely associated with neuronal death. Inhibiting nNOS can protect neurons from cell death caused by peroxynitrite cytotoxicity (Faglioni et al. 2014). Excessive immune responses mediated by P‐P65 can exacerbate neuroinflammation and damage neural tissues, thereby affecting various neurological diseases such as neuritis and retinitis (Xu et al. 2024).In this study, we observed that CUR can inhibit the inflammation and reduce the MN death.

GAP‐43, a specific phosphoprotein, is widely used as a biomarker for neuronal development, regeneration, and synaptic plasticity (Liu et al. 2023).The neural cell adhesion molecule L1 not only promotes neuronal adhesion and cell migratio, but also supports axonal growth and guidance, as well as myelination and synaptic plasticity (He et al. 2022). HMCGR can promote the myelination of axons. NGFR is considered one of the inhibitors of axonal regeneration in the central nervous system and is found to have increased expression in an autoimmune demyelination mouse model (Theotokis et al. 2016). Abnormal expression of POU3F1 leads to reduced myelination in peripheral nerves and axonal loss (Ryu et al. 2007). In our study, we found that CUR can reduce the MN death and promote the remyelination.

After a motor nerve injury, axoplasmic transport between the nerve and its target muscle is completely interrupted, causing the target muscle to lose neural trophic support. This leads to rapid muscle atrophy and the development of muscle fibrosis (Xie et al. 2019). The rate of nerve fiber regeneration is slow, and often the material basis for muscle cell regeneration is lost before reinnervation occurs, severely affecting the recovery of motor function after nerve reimplantation surgery. In our study, we found that after CUR can reduce muscle atrophy.

All in all, this study indicated that CUR can facilitate the recovery of motor function in rats following BPRA via improving the survival of MNs and promoting the remyelination of axonal myelin and reducing muscle atrophy. Overall, curcumin may serve as a potential therapeutic agent for treating BPRA in the clinic.

## Author Contributions

**Sijing Li**: investigation, data curation, and methodology. **Bing He**: investigation. **Guijuan Zhou**: investigation. **Lin Wu**: investigation. **Xuanwei Wen**: investigation. **Limin Deng**: investigation. **Shudong Lin**: investigation. **Guozhi Liu**: investigation. **Shuangxi Chen**: writing – original draft, investigation, and funding acquisition. **Zijian Xiao**: conceptualization, funding acquisition, writing – review and editing, and methodology.

## Conflicts of Interest

The authors declare no conflicts of interest.

## Peer Review

The peer review history for this article is available at https://publons.com/publon/10.1002/brb3.70728

## Data Availability

The data that support the findings of this study are available from the corresponding author upon reasonable request.
